# Supplementary material for: A Likelihood Approach for Real-Time Calibration of Stochastic Compartmental Epidemic Models
Source: PLoS Comput Biol. 2017 Jan 17;13(1):e1005257. doi: 10.1371/journal.pcbi.1005257 (PMC5240920; doi:10.1371/journal.pcbi.1005257)
Supplement: S1 File — (TAR.GZ) [file pcbi.1005257.s014.tar.gz › HSPH_Online-SI-Revision/Additions/MSS11/n100-extreme_noisy/n100-extreme_noisy_NEW_table8.pdf]

1	R0	{2.76052, 16.3913, 0.1}	{2.98125, 18.4749, 2.}	{{2.98125, 18.4749, 2.}, {2.98125, 18.4749, 2.}}	{7.99606, 12.7113, 1900.}	inf	3200.	4248.15	{4195.58, 4301.87}	32.7547	0.123031	newly	1200.81	1388.66	{1344., 1433.}	15.6432	0.0629874	Reff	0.245824	0.212391	{0.211579, 0.213088}	13.6006	ar	92 090.	93 905.8	{93 853., 93 968.}	1.9718
2	R0	{2.18202, 15.1734, 3.}	{2.22867, 15.6411, 3.}	{{2.22867, 15.6411, 3.}, {2.22867, 15.6411, 3.}}	{2.13793, 3.08213, 0.}	inf	3327.	3432.58	{3382.93, 3493.38}	3.17338	0.0135353	newly	1337.	1341.42	{1293., 1384.}	2.11369	0.00915651	Reff	0.385257	0.377908	{0.377166, 0.378692}	1.90762	ar	84 264.	84 936.	{84 844., 85 025.}	0.797517
3	R0	{2.57891, 12.5971, 1.}	{2.53075, 11.6597, 0.}	{{2.53075, 11.6597, 0.}, {2.53075, 11.6597, 0.}}	{1.86748, 7.44128, 100.}	inf	1362.	1044.42	{1002.95, 1077.17}	23.3169	0.115444	newly	609.	505.75	{477., 534.}	16.954	0.0810931	Reff	0.262765	0.271324	{0.270865, 0.271791}	3.25724	ar	90 302.	89 661.	{89 612., 89 700.}	0.709862
4	R0	{2.5856, 9.28508, 0.1}	{2.93211, 10.3741, 0.02}	{{2.93906, 10.3908, 0.}, {2.93906, 10.3908, 0.}}	{13.4468, 11.7429, 116.}	inf	433.	566.729	{541.865, 593.204}	30.8843	0.11651	newly	254.	296.3	{278., 315.}	16.6535	0.0662334	Reff	0.254526	0.192818	{0.191201, 0.191948}	24.3373	ar	90 312.	93 548.8	{93 589., 93 639.}	3.59969
5	R0	{2.4406, 12.1341, 1.}	{2.58042, 13.7525, 1.98}	{{2.58092, 13.7736, 2.}, {2.58092, 13.7736, 2.}}	{5.72894, 13.4157, 100.}	inf	1891.	2600.37	{2559.43, 2656.01}	37.7293	0.138941	newly	894.	1107.41	{1066., 1148.}	24.0369	0.09337	Reff	0.297534	0.275231	{0.274634, 0.275817}	7.49588	ar	88 597.	90 260.8	{90 186., 90 333.}	1.87789
6	R0	{2.72155, 15.4094, 2.}	{2.93371, 17.1903, 1.}	{{2.93371, 17.1903, 1.}, {2.93371, 17.1903, 1.}}	{7.79541, 11.5572, 50.}	inf	3044.	3787.78	{3731.89, 3839.83}	24.4342	0.094911	newly	1132.	1319.35	{1273., 1364.}	16.5504	0.0663632	Reff	0.252533	0.216671	{0.215737, 0.217358}	14.2008	ar	91 682.	93 569.7	{93 510., 93 633.}	2.05899
7	R0	{2.63781, 14.3935, 0.1}	{2.46794, 14.1192, 0.}	{{2.46794, 14.1192, 0.}, {2.46794, 14.1192, 0.}}	{6.44, 1.90541, 100.}	inf	2374.	2415.46	{2365.43, 2470.78}	2.00002	0.00856759	newly	968.	1012.15	{970., 1048.}	4.72417	0.019913	Reff	0.252043	0.299524	{0.298798, 0.300128}	18.8384	ar	91 192.	88 839.6	{88 775., 88 912.}	2.57967
8	R0	{2.82391, 16.104, 1.}	{2.93371, 17.1903, 1.}	{{2.93371, 17.1903, 1.}, {2.93371, 17.1903, 1.}}	{3.88808, 6.74551, 0.}	inf	3239.	3790.21	{3725.86, 3844.77}	17.018	0.0682239	newly	1178.	1320.67	{1266., 1371.}	12.1112	0.0494783	Reff	0.232408	0.216555	{0.215769, 0.217353}	6.82114	ar	92 664.	93 577.7	{93 513., 93 636.}	0.985992
9	R0	{2.80645, 14.6457, 0.1}	{2.7499, 15.3124, 0.}	{{2.7499, 15.3124, 0.}, {2.7499, 15.3124, 0.}}	{2.0151, 4.55228, 100.}	inf	2875.	3570.02	{3518.87, 3611.07}	24.1745	0.0940102	newly	1160.	1377.06	{1340., 1417.}	18.7121	0.0743875	Reff	0.229399	0.248858	{0.248058, 0.249756}	8.48255	ar	92 567.	92 032.5	{91 971., 92 094.}	0.577387
10	R0	{1.86213, 5.4271, 0.1}	{1.81768, 5.68021, 1.}	{{1.81768, 5.68021, 1.}, {1.81768, 5.68021, 1.}}	{2.38702, 4.66374, 900.}	inf	95.	106.887	{91.498, 122.682}	15.1901	0.0607523	newly	92.	98.26	{84., 113.}	11.9348	0.0493078	Reff	0.460598	0.476106	{0.4757, 0.476413}	3.36697	ar	75 362.	73 901.7	{73 849., 73 955.}	1.93766
11	R0	{2.27349, 14.6338, 1.}	{2.20937, 14.0906, 2.97}	{{2.20345, 14.058, 3.}, {2.20345, 14.058, 3.}}	{3.15634, 3.92195, 197.}	inf	2757.	2816.83	{2755.95, 2876.76}	2.24076	0.00957532	newly	1137.	1203.66	{1162., 1253.}	6.01231	0.0252064	Reff	0.351982	0.375938	{0.376684, 0.378327}	7.294	ar	85 805.	84 537.1	{84 342., 84 537.}	1.62104
12	R0	{2.77261, 14.1894, 0.1}	{2.73108, 15.6124, 0.}	{{2.73108, 15.6124, 0.}, {2.73108, 15.6124, 0.}}	{1.4977, 10.0288, 100.}	inf	2393.	3150.84	{3113.58, 3189.53}	31.6692	0.119461	newly	959.	1200.76	{1163., 1242.}	25.2096	0.097508	Reff	0.226855	0.248625	{0.247952, 0.249231}	9.59666	ar	92 508.	91 859.3	{91 809., 91 921.}	0.701215
13	R0	{2.65402, 19.3935, 2.}	{2.76795, 19.6041, 1.}	{{2.76795, 19.6041, 1.}, {2.76795, 19.6041, 1.}}	{4.2927, 1.08616, 50.}	inf	6091.	6484.19	{6422.3, 6535.58}	6.45534	0.0271562	newly	2008.	2037.55	{1983., 2091.}	2.13197	0.00914923	Reff	0.292659	0.273453	{0.272406, 0.27452}	6.56251	ar	91 184.	92 206.9	{92 114., 92 290.}	1.12183
14	R0	{1.75297, 4.45354, 0.1}	{2.22376, 9.35432, 0.}	{{2.22376, 9.35432, 0.}, {2.22376, 9.35432, 0.}}	{26.8565, 110.042, 100.}	inf	33.	810.003	{775.033, 843.65}	2354.56	1.38976	newly	37.	478.57	{453., 506.}	1193.43	1.11127	Reff	0.50468	0.346383	{0.345819, 0.346932}	31.3657	ar	71 263.	84 847.7	{84 787., 84 894.}	19.0628
15	R0	{1.99928, 7.1501, 0.1}	{2.13505, 8.31782, 0.}	{{2.13505, 8.31782, 0.}, {2.13505, 8.31782, 0.}}	{6.79086, 16.3315, 100.}	inf	273.	394.188	{366.948, 418.711}	44.3912	0.159018	newly	197.	257.42	{233., 282.}	30.7107	0.115236	Reff	0.411872	0.367715	{0.367284, 0.368149}	10.7211	ar	79 602.	83 009.6	{82 958., 83 065.}	4.28075
16	R0	{2.67398, 10.3134, 1.}	{2.64721, 11.9965, 0.}	{{2.64721, 11.9965, 0.}, {2.64721, 11.9965, 0.}}	{1.00103, 16.3197, 100.}	inf	624.	1352.49	{1318.84, 1391.69}	116.745	0.335852	newly	342.	638.24	{608., 667.}	86.6199	0.270631	Reff	0.237957	0.24948	{0.248951, 0.250037}	4.84239	ar	91 289.	91 010.3	{90 958., 91 064.}	0.305272
17	R0	{2.44634, 16.3226, 0.1}	{2.73517, 19.6025, 2.}	{{2.73517, 19.6025, 2.}, {2.73517, 19.6025, 2.}}	{11.8066, 20.0943, 1900.}	inf	3997.	5464.41	{5413.19, 5524.9}	36.7128	0.135796	newly	1461.	1709.85	{1659., 1750.}	17.0329	0.0682128	Reff	0.317486	0.27002	{0.26902, 0.270943}	14.9505	ar	88 710.	91 890.5	{91 811., 91 972.}	3.58523
18	R0	{2.02168, 10.1676, 0.1}	{2.02235, 10.6804, 0.}	{{2.02235, 10.6804, 0.}, {2.02235, 10.6804, 0.}}	{0.0333691, 5.04351, 100.}	inf	1489.	1800.69	{1754.37, 1842.07}	20.9326	0.0824693	newly	847.	983.04	{947., 1017.}	16.0614	0.0644965	Reff	0.420772	0.424176	{0.42363, 0.424687}	0.809033	ar	80 299.	80 294.2	{80 199., 80 385.}	0.076651
19	R0	{2.89969, 15.009, 0.1}	{2.93371, 17.1903, 1.}	{{2.93371, 17.1903, 1.}, {2.93371, 17.1903, 1.}}	{1.17309, 14.5333, 900.}	inf	2686.	3785.05	{3731.84, 3838.27}	40.9179	0.148945	newly	1026.	1317.61	{1274., 1360.}	28.422	0.108511	Reff	0.211938	0.21663	{0.215914, 0.217349}	2.21356	ar	93 349.	93 572.7	{93 511., 93 633.}	0.239595
20	R0	{2.68933, 7.34928, 0.1}	{2.93906, 10.3908, 0.}	{{2.93906, 10.3908, 0.}, {2.93906, 10.3908, 0.}}	{9.28584, 41.385, 100.}	inf	136.	569.56	{543.902, 592.023}	318.794	0.621763	newly	80.	296.28	{275., 315.}	270.35	0.567997	Reff	0.227975	0.191481	{0.191093, 0.191891}	16.0077	ar	91 574.	93 619.3	{93 591., 93 644.}	2.23354
21	R0	{2.15352, 9.28074, 0.1}	{2.38758, 11.1331, 1.}	{{2.38758, 11.1331, 1.}, {2.38758, 11.1331, 1.}}	{10.8685, 19.9593, 900.}	inf	838.	1251.4	{1209.74, 1287.01}	49.3312	0.174036	newly	504.	639.	{614., 669.}	26.7857	0.102799	Reff	0.358733	0.307169	{0.306617, 0.307636}	14.3741	ar	83 845.	87 667.2	{87 614., 87 727.}	4.55865
22	R0	{2.21953, 9.04387, 0.1}	{2.2983, 8.81055, 0.}	{{2.2983, 8.81055, 0.}, {2.2983, 8.81055, 0.}}	{3.54873, 2.57984, 100.}	inf	694.	477.363	{455.712, 501.801}	31.2157	0.162846	newly	414.	292.84	{270., 313.}	29.2657	0.151085	Reff	0.343938	0.321996	{0.321508, 0.32242}	6.37983	ar	84 856.	86 211.6	{86 171., 86 253.}	1.59753
23	R0	{2.21987, 14.5347, 1.}	{2.76795, 19.6041, 1.}	{{2.76795, 19.6041, 1.}, {2.76795, 19.6041, 1.}}	{24.6897, 34.8782, 0.}	inf	2885.	4993.35	{4940.84, 5048.74}	73.0799	0.238232	newly	1192.	1564.54	{1519., 1602.}	31.2534	0.118006	Reff	0.366057	0.258375	{0.25755, 0.259261}	29.4167	ar	85 067.	92 207.2	{92 126., 92 274.}	8.39358
24	R0	{2.84268, 17.9659, 1.}	{2.56757, 17.005, 0.}	{{2.56757, 17.005, 0.}, {2.56757, 17.005, 0.}}	{9.67771, 5.34863, 100.}	inf	4541.	4127.76	{4065.68, 4187.26}	9.1002	0.0414676	newly	1477.	1470.76	{1425., 1516.}	2.07448	0.00904008	Reff	0.242139	0.293784	{0.292892, 0.294634}	21.3286	ar	92 836.	90 115.7	{90 032., 90 212.}	2.93023
25	R0	{2.52706, 10.3679, 0.1}	{2.57307, 13.9636, 0.}	{{2.27199, 11.6669, 0.}, {2.7499, 15.3124, 0.}}	{9.28994, 34.681, 100.}	inf	746.	2145.99	{1469.48, 2575.21}	187.666	0.445058	newly	432.	888.15	{713., 1012.}	105.59	0.30848	Reff	0.269107	0.276883	{0.238889, 0.340884}	16.7858	ar	89 623.	89 716.7	{85 699., 92 095.}	3.30497
26	R0	{2.62835, 19.1088, 1.}	{2.73517, 19.6025, 2.}	{{2.73517, 19.6025, 2.}, {2.73517, 19.6025, 2.}}	{4.06417, 2.58367, 100.}	inf	5501.	5465.88	{5405.6, 5517.91}	0.816924	0.00356868	newly	1758.	1717.36	{1673., 1766.}	2.56997	0.0113772	Reff	0.292378	0.269937	{0.269142, 0.270651}	7.67508	ar	90 908.	91 898.	{91 824., 91 982.}	1.08896
27	R0	{2.76831, 19.4155, 2.}	{2.42687, 16.0321, 3.}	{{2.42687, 16.0321, 3.}, {2.42687, 16.0321, 3.}}	{12.3339, 17.4265, 50.}	inf	4502.	3195.97	{3145.55, 3242.41}	29.01	0.148834	newly	1418.	1203.66	{1166., 1238.}	15.1157	0.0713028	Reff	0.252857	0.318403	{0.317707, 0.319119}	25.9221	ar	92 175.	88 254.4	{88 163., 88 344.}	4.25341
28	R0	{2.96609, 16.8903, 1.}	{2.59907, 13.5234, 2.}	{{2.59907, 13.5234, 2.}, {2.59907, 13.5234, 2.}}	{12.3737, 19.9337, 100.}	inf	3132.	2082.36	{2039.29, 2128.66}	33.5134	0.177335	newly	1123.	900.01	{866., 936.}	19.8566	0.0963265	Reff	0.198639	0.266301	{0.265661, 0.266979}	34.0627	ar	94 024.	90 471.6	{90 411., 90 540.}	3.77814
29	R0	{2.17046, 14.39, 0.1}	{2.15014, 13.8434, 1.}	{{2.1255, 13.6009, 1.}, {2.315, 15.4658, 1.}}	{2.66786, 5.74245, 900.}	inf	3337.	3228.91	{3090.73, 3728.1}	6.58543	0.0288212	newly	1397.	1404.17	{1360., 1451.}	2.17967	0.00938398	Reff	0.390705	0.400927	{0.356715, 0.408489}	4.91511	ar	84 001.	83 275.6	{82 684., 86 434.}	1.64043
30	R0	{2.88772, 12.4647, 1.}	{2.79531, 12.1249, 2.}	{{2.79531, 12.1249, 2.}, {2.79531, 12.1249, 2.}}	{3.20015, 2.72599, 100.}	inf	1402.	1409.3	{1371.71, 1443.51}	1.57275	0.00679612	newly	651.	654.13	{620., 690.}	3.1874	0.0137837	Reff	0.203498	0.221746	{0.221156, 0.222352}	8.9674	ar	93 273.	92 460.9	{92 420., 92 501.}	0.870713
31	R0	{2.46831, 8.14308, 0.1}	{2.17292, 6.16453, 0.}	{{2.17292, 6.16453, 0.}, {2.17292, 6.16453, 0.}}	{11.9675, 24.2973, 100.}	inf	300.	106.969	{93.8274, 120.418}	64.3436	0.449825	newly	189.	86.11	{72., 99.}	54.4392	0.344613	Reff	0.280894	0.352853	{0.352618, 0.353107}	25.6179	ar	88 762.	83 818.4	{83 794., 83 844.}	5.56955
32	R0	{1.93236, 7.38987, 0.1}	{1.74592, 5.58477, 0.}	{{1.74592, 5.58477, 0.}, {1.74592, 5.58477, 0.}}	{9.64841, 24.4266, 100.}	inf	389.	186.222	{164.901, 204.666}	52.128	0.321362	newly	288.	172.73	{151., 196.}	40.0243	0.224002	Reff	0.437467	0.50791	{0.507515, 0.508288}	16.1025	ar	77 654.	71 105.9	{71 045., 71 163.}	8.43237
33	R0	{2.82251, 17.6954, 1.}	{2.51743, 15.191, 3.}	{{2.51743, 15.191, 3.}, {2.51743, 15.191, 3.}}	{10.8089, 14.1528, 200.}	inf	4866.	3696.91	{3636.77, 3742.92}	24.0257	0.119358	newly	1670.	1452.61	{1404., 1503.}	13.0174	0.0606892	Reff	0.244147	0.301085	{0.300268, 0.301829}	23.321	ar	92 745.	89 488.4	{89 412., 89 579.}	3.51129
34	R0	{1.87083, 12.3994, 1.}	{2.07206, 15.0886, 4.}	{{2.07206, 15.0886, 4.}, {2.07206, 15.0886, 4.}}	{10.7562, 21.6882, 300.}	inf	3095.	4218.47	{4150.86, 4277.58}	36.2997	0.134466	newly	1528.	1719.17	{1675., 1762.}	12.5111	0.0511112	Reff	0.498595	0.443706	{0.442684, 0.444705}	11.0088	ar	76 282.	81 548.5	{81 400., 81 685.}	6.90399
35	R0	{2.95779, 16.3003, 1.}	{2.5684, 13.7324, 1.}	{{2.5684, 13.7324, 1.}, {2.5684, 13.7324, 1.}}	{13.1649, 15.7535, 0.}	inf	3818.	2655.53	{2595.82, 2702.36}	30.4471	0.157726	newly	1428.	1133.54	{1090., 1176.}	20.6204	0.100443	Reff	0.209086	0.27845	{0.277754, 0.279131}	33.1747	ar	93 873.	90 113.9	{90 040., 90 193.}	4.00443
36	R0	{2.18718, 14.261, 3.}	{2.2143, 14.4309, 4.}	{{2.2143, 14.4309, 4.}, {2.2143, 14.4309, 4.}}	{1.24015, 1.19125, 33.3333}	inf	3033.	3329.72	{3284.53, 3382.41}	9.78292	0.0405078	newly	1295.	1390.19	{1349., 1434.}	7.35058	0.0306929	Reff	0.381269	0.381059	{0.380324, 0.381889}	0.135125	ar	84 379.	84 656.9	{84 549., 84 755.}	0.3293
37	R0	{2.44912, 9.31118, 0.1}	{2.19024, 8.25876, 1.}	{{2.19024, 8.25876, 1.}, {2.19024, 8.25876, 1.}}	{10.5704, 11.3028, 900.}	inf	559.	439.483	{416.354, 462.431}	21.3804	0.104865	newly	306.	285.43	{262., 307.}	7.15359	0.0328048	Reff	0.289559	0.351798	{0.35139, 0.352316}	21.4941	ar	88 442.	84 168.7	{84 124., 84 215.}	4.83179
38	R0	{2.05949, 5.72514, 0.1}	{1.9862, 6.28417, 0.}	{{1.9862, 6.28417, 0.}, {1.9862, 6.28417, 0.}}	{3.55866, 9.76441, 100.}	inf	81.	144.154	{127.276, 161.774}	77.9682	0.24858	newly	76.	117.73	{99., 133.}	54.9079	0.187616	Reff	0.380141	0.413108	{0.412799, 0.413391}	8.67248	ar	81 587.	79 302.5	{79 265., 79 340.}	2.8001
39	R0	{1.75505, 8.52404, 0.1}	{1.87584, 10.2502, 2.}	{{1.87584, 10.2502, 2.}, {1.87584, 10.2502, 2.}}	{6.88266, 20.2508, 1900.}	inf	1029.	1612.64	{1565.28, 1656.87}	56.7195	0.195025	newly	719.	924.38	{887., 966.}	28.5647	0.108892	Reff	0.527357	0.477622	{0.47681, 0.478357}	9.43115	ar	70 925.	75 931.5	{75 824., 76 064.}	7.05879
40	R0	{2.24724, 13.7861, 1.}	{2.2875, 14.5984, 3.99}	{{2.28604, 14.5901, 4.}, {2.28604, 14.5901, 4.}}	{1.79131, 5.89238, 299.}	inf	2420.	2618.98	{2572.23, 2662.09}	8.22225	0.0342042	newly	1045.	1075.44	{1022., 1118.}	3.56364	0.0151433	Reff	0.360008	0.349739	{0.349398, 0.350739}	2.85237	ar	85 257.	86 020.8	{85 913., 86 069.}	0.895856
41	R0	{1.77635, 8.62406, 0.1}	{1.96373, 10.9366, 4.}	{{1.96373, 10.9366, 4.}, {1.96373, 10.9366, 4.}}	{10.5483, 26.8153, 3900.}	inf	882.	1594.12	{1548.81, 1639.38}	80.7396	0.256949	newly	608.	859.12	{823., 893.}	41.3026	0.149915	Reff	0.494927	0.442631	{0.441908, 0.44327}	10.5663	ar	72 941.	78 662.7	{78 557., 78 760.}	7.84429
42	R0	{1.80397, 5.4932, 1.}	{1.81768, 5.68021, 1.}	{{1.81768, 5.68021, 1.}, {1.81768, 5.68021, 1.}}	{0.760022, 3.40431, 0.}	inf	102.	102.779	{89.5022, 117.682}	8.49111	0.036725	newly	82.	92.36	{77., 109.}	15.9268	0.0633034	Reff	0.48442	0.476108	{0.475799, 0.476356}	1.71588	ar	73 232.	73 902.4	{73 865., 73 935.}	0.915474
43	R0	{2.83096, 16.7077, 0.1}	{2.48143, 15.7026, 0.}	{{2.48143, 15.7026, 0.}, {2.48143, 15.7026, 0.}}	{12.3469, 6.01581, 100.}	inf	3887.	4005.22	{3944.85, 4058.18}	3.04215	0.01299	newly	1377.	1534.17	{1493., 1576.}	11.4139	0.0468185	Reff	0.237546	0.313617	{0.312734, 0.314472}	32.0238	ar	92 740.	89 012.1	{88 926., 89 093.}	4.01968
44	R0	{2.73678, 11.6021, 1.}	{2.99939, 13.7076, 1.}	{{2.99939, 13.7076, 1.}, {2.99939, 13.7076, 1.}}	{9.5957, 18.1478, 0.}	inf	851.	1489.02	{1456.15, 1524.52}	74.9724	0.242899	newly	393.	622.05	{594., 648.}	58.2824	0.199182	Reff	0.225237	0.188677	{0.188157, 0.189231}	16.232	ar	92 003.	94 044.6	{93 999., 94 081.}	2.21907
45	R0	{2.14235, 6.98601, 0.1}	{2.13505, 8.31782, 0.}	{{2.13505, 8.31782, 0.}, {2.13505, 8.31782, 0.}}	{0.340824, 19.0639, 100.}	inf	172.	393.304	{368.969, 424.305}	128.665	0.358664	newly	135.	255.52	{236., 276.}	89.2741	0.276226	Reff	0.36707	0.367705	{0.367239, 0.368123}	0.175901	ar	82 974.	83 004.2	{82 955., 83 044.}	0.0466411
46	R0	{2.23409, 9.21289, 0.1}	{2.53075, 11.6597, 0.}	{{2.53075, 11.6597, 0.}, {2.53075, 11.6597, 0.}}	{13.2788, 26.5587, 100.}	inf	866.	1615.85	{1579.37, 1664.14}	86.5875	0.27079	newly	520.	789.17	{752., 825.}	51.7635	0.180913	Reff	0.341659	0.276816	{0.27604, 0.277473}	18.9791	ar	85 163.	89 652.6	{89 588., 89 708.}	5.27182
47	R0	{2.12258, 8.15265, 0.1}	{2.19024, 8.25876, 1.}	{{2.19024, 8.25876, 1.}, {2.19024, 8.25876, 1.}}	{3.18751, 1.30154, 900.}	inf	433.	441.112	{415.633, 467.33}	4.09324	0.0174611	newly	280.	287.1	{265., 309.}	5.45	0.0230835	Reff	0.375293	0.351693	{0.351245, 0.352108}	6.28846	ar	82 690.	84 180.6	{84 138., 84 222.}	1.80264
48	R0	{2.71771, 10.4636, 0.1}	{2.74056, 10.6768, 0.}	{{2.74056, 10.6768, 0.}, {2.74056, 10.6768, 0.}}	{0.840716, 2.03712, 100.}	inf	924.	831.264	{807.648, 853.055}	10.0363	0.0460409	newly	498.	426.15	{405., 448.}	14.4277	0.0680226	Reff	0.232147	0.227102	{0.226628, 0.227594}	2.17313	ar	91 716.	91 950.8	{91 916., 91 986.}	0.255964
49	R0	{2.44369, 6.66477, 0.1}	{2.77394, 8.24535, 0.}	{{2.77394, 8.24535, 0.}, {2.77394, 8.24535, 0.}}	{13.5144, 23.7155, 100.}	inf	49.	157.993	{142.656, 172.845}	222.435	0.507298	newly	43.	97.56	{87., 109.}	126.884	0.353812	Reff	0.273693	0.215777	{0.215476, 0.216002}	21.1608	ar	88 827.	92 265.2	{92 247., 92 283.}	3.87071
50	R0	{2.77383, 17.9775, 0.1}	{2.66269, 17.3235, 0.92}	{{2.64334, 17.2462, 1.}, {2.64334, 17.2462, 1.}}	{4.64927, 3.84717, 836.}	inf	3843.	3585.36	{3536.37, 3631.59}	6.70412	0.0301624	newly	1236.	1250.82	{1206., 1294.}	2.47573	0.0106623	Reff	0.244319	0.267177	{0.269676, 0.271795}	10.7211	ar	92 334.	91 145.7	{90 906., 91 061.}	1.43713
